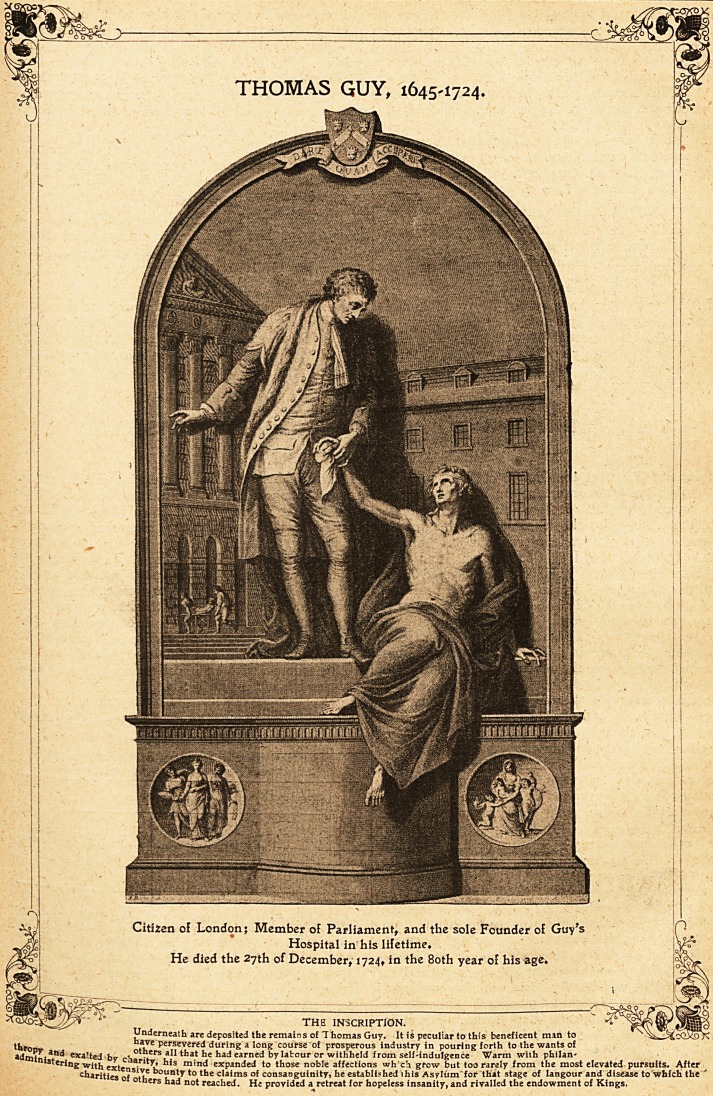# The Chapel of Guy's Hospital

**Published:** 1917-06-23

**Authors:** 


### THE CHAPEL OF GUY'S HOSPITAL.

Guy's men have deservedly had the reputation throughout the world of being men of action and resource, who could be safely trusted in an emergency to rise to it, and it is felt that whatever his feelings might be no man who has had the advantage of the teaching and training at Guy's can fail to do his duty to the utmost of his powers and ability. "Thorough" has been written across the entrance to Guy's Hospital, through decades of years. Of all the hospitals in Great Britain, Guy's to-day occupies a unique position in the eyes of hospital students, and of inquirers after knowledge of the underlying causes which have most contributed to produce the maximum of result in the building up of the modern hospital. This is especially true where a great hospital, like that founded by Thomas Guy in 1724, rests upon an ancient foundation which has been modernised, brought up to date, and perfected by a continuous output of administrative, technical, and all-embracing knowledge on the part of those who have been mainly responsible for the wonderful changes and developments which have taken place in its buildings and organisation since its reconstruction commenced in 1895. If anyone doubts this, especially if he is an old Guy's man, let him take an opportunity of visiting Guy's, passing through the entrance quadrangle and up the steps to the middle of the colonnade and look back towards the entrance and then towards the park. The governors, superintendent, and architect are to be congratulated upon the strikingly effective changes and attractiveness produced in the colonnade recently, which, with Rust's vitreous mosaic pavement given by Mr. Cosmo Bonsor in 1899, constitute it a most winsome and beautiful feature of old Guy's rejuvenated and largely rebuilt.

The chapel occupies the centre of the buildings on the right of the quadrangle as the visitor approaches the hospital from St. Thomas's Street. Externally it presents few ecclesiastical features, but internally its atmosphere and decorations, together with its monuments and tablets, though few in number, present many interesting features, and several might well be taken as examples to follow by other hospitals whose managers recognise the importance of making the chapel the centre of life for all workers, and especially for those who spend years in service for a great institution. Amongst the most notable features are the Panels and Mosaics, the former in oak of very attractive and restful designs, the latter Vitreous Mosaics by Powell and Sons, of Tudor Street, Whitefriars, which are in some lights, especially when the electric lamps are turned on, strikingly beautiful. These panels and mosaics, placed on the walls under the galleries, were added by subscriptions in 1903 as a memorial "to those men and women who have died in the medical service of Guy's Hospital since 1867." The memorial consists of oak panels, on which the names appear, and Opus Sectile Mosaics, designed and executed by James Powell and Sons. The seven mosaics cost rather more than £350, and the completed oak panels another £50. The side panels of the triptych over the Communion Table, representing St. Barnabas and St. Luke, were completed in May 1899, at a cost of £160, by Percy Bacon and Brothers. The chalice and paten were presented for use in the chapel by the family of the late Sir Thomas Stevenson.

It is interesting to note that up to the end of 1901 the choir consisted of boys, but at Christmas in that year, upon the appointment of Mr. Henry Taylor as organist, the nurses took the place of the boys, and in November 1902 the students of the hospital formed a male choir, first singing in chapel on November 9, 1902. Owing to the war the male choir are absent and greatly missed at present, because of the brightness and life they gave to the music. It might be well to appoint a sister as Warden, seeing that of the three young males present in the chapel during morning service, one of them never stood once, and two of them remained sitting during most of the service, including the Creed, a display of irreverence which the presence of a Warden would no doubt have prevented. The Lessons were indistinctly read and scarcely audible in the back seats, to the loss of those present who tried to follow. The Chaplain is the Bishop of the Diocese. £350 per annum is paid to Acting Chaplains. There is a Chapel Committee which consists of the Chaplains, the superintendent, the matron, and the honorary secretary as *ex-officio* members, together with not less than twelve nor more than fifteen elected members appointed annually at a meeting of the congregation, held in the summer. The Committee's resolutions affect (*a*) the constitution of the Committee; (*b*) the disposal of offertories and other gifts; (*c*) the decorations and music; (*d*) hours of service; (*e*) seating and other arrangements for the convenience of the congregation; and (*f*) alteration of rules. No rule passed by the Chapel Committee "shall" come into operation until it has received the sanction of the House Committee. Electric light was installed in 1899, and in November 1900 the old stone floor of the chapel was replaced by mosaic at a cost of £58, when an oak enclosure was sanctioned for the enlargement of the vestry, the work being carried out by the works department of the hospital at a cost of £65.

The sick at Guy's Hospital have every facility afforded to them for receiving the visits of the ministers of their respective denominations, and due respect is paid to the religious convictions of all who are inmates of the institution. Guy's Hospital Sunday Preachers are provided for the services in the wards. The Sunday Preachers consist of a band of laymen, about thirty in number, the chairman being Mr. F. W. Cowley, of Sydenham, who visit the wards every Sunday evening. The visit begins at 5.30 and ends at 7 P.M., during which time "each company of two workers visits two wards, the length of each visit being about forty minutes." The workers are generally a senior and a junior—the former reads a portion of Scripture and gives a ten-minutes Gospel address. The latter reads the prayers, consisting of the General Confession, the Collect for the day, the Lord's Prayer, a prayer for the hospital, and the prayer of St. Chrysostom. Hymns are sung, and the whole service lasts about twenty-five minutes, the remainder of the time being spent in visiting the beds and distributing to patients suitable tracts, most usually "Friendly Greetings," a Religious Tract Society publication.

A marble panel bears the name of the late superintendent, Dr. John G. Steele, who died in 1892, aged seventy-one, after forty-one years' service. A marble tablet to the left of the pew in the gallery occupied by the superintendent testifies to Dr. Steele's "kindness of heart and large experience "which enabled him to perform so faithfully the "manifold duties of his office, which will be long "remembered by the governors and staff who "caused this tablet to be erected." Dr. Steele had a multitude of friends, his friendship was treasured by a number of his fellow-workers, and his death was sincerely mourned by many representative people, including his colleagues, hospital workers, governors, and philanthropists generally.

### MEMORIALS TO GOVERNORS.

Amongst the governors to whom memorials have been placed on the walls of the chapel are the Right Hon. W. E. Gladstone, six times Prime Minister and for sixty years a governor, who took a continuous interest in the hospital and never failed to render it personal service when opportunity offered. He was born in 1809 and died in 1898. The memorial to Rodolph Alexander Hankey records service from 1893 to 1906, and ends with the words, "He loved the hospital." "In Memory of Marcus Trevelyan Martin. He was a governor of Guy's Hospital from 1899 to the time of his death in 1908. A friend to the sick and needy." That to Mr. Alfred Beit, "In grateful memory 1896 to 1906," is the simplest, best-executed, and most attractive memorial plate we have ever seen. One memorial, for some unexplained reason, occupies a prominent place outside the chapel in the passage facing the entrance. It was originally decorative in form, but has suffered from the weather. This is to two governors, the

### THOMAS GUY, 1645-1724.

Citizen of London; Member of Parliament, and the sole Founder of Guy's Hospital in his lifetime.
He died the 27th of December, 1724, in the 80th year of his age.

THE INSCRIPTION.

Underneath are deposited the remains of Thomas Guy. It is peculiar to this beneficent man to have persevered during a long course of prosperous industry in pouring forth to the wants of others all that he had earned by labour or withheld from self-indulgence. Warm with philanthropy and exalted by charity, his mind expanded to those noble affections which grow but too rarely from the most elevated pursuits. After administering with extensive bounty to the claims of consanguinity, he established this Asylum for that stage of langour and disease to which the charities of others had not reached. He provided a retreat for hopeless insanity, and rivalled the endowment of Kings.

Rev. Arthur Cazenove, son, and Philip Cazenove his father. The latter was one of the most popular and influential members of the Stock Exchange in his day.

BENEFACTORS.

(1) "Miss Louisa Anne Williams, a generous benefactor (£30,000) to this hospital. Died 1910, aged 90."

(2) "This bust was placed here by the Governors to perpetuate the remembrance of Matthew Whiting, a generous benefactor of this hospital, 1901. Lord, keep my memory green."

PERSONAL SERVICE.

"Clemence Margaret Cotton, who devoted herself to the work of this hospital from May 1892 to her death in 1902. Inasmuch as——"

SISTERS AND NURSES.

In the years that have passed many noble acts have been performed and much and great personal service has been rendered by women to the sick within the walls of Guy's. We have selected three instances to illustrate the permanent value and never-failing interest and vitality of memorials to departed workers within a hospital chapel. The first is Sister "Clara Faithful Lumley, Sister of Accident Ward. A short career of devotion and fidelity. She died from small-pox caught in the discharge of her duty, March 1892, aged 33." Miss Lumley was one of the most popular and devoted of sisters. Her memory is still alive, and her works do follow her.

A NURSE.

"To the memory of a brave and unselfish woman, Alice Liddon, nurse of this hospital, who was faithful to her duty unto death. February 22, 1888, aged 26."

NIGHT NURSE.

"Placed by Past and Present Students in memory of Mrs. Keogh, for many years Night Nurse in the Surgery of this hospital. Died December 11, 1897, aged 65 years."

AND A FORMER MATRON.

Finally, it may interest older workers to reproduce here "A token of esteem and affection by a few friends and fellow-workers of Margaret Elizabeth Field (*née* Burt), matron November 1879 to November 1882. Died 1892. She hath done what she could."

## Figures and Tables

**Figure f1:**